# Association of mitochondrial DNA copy number with metabolic syndrome and type 2 diabetes in 14 176 individuals

**DOI:** 10.1111/joim.13242

**Published:** 2021-02-20

**Authors:** F. Fazzini, C. Lamina, A. Raftopoulou, A. Koller, C. Fuchsberger, C. Pattaro, F. M. Del Greco, P. Döttelmayer, L. Fendt, J. Fritz, H. Meiselbach, S. Schönherr, L. Forer, H. Weissensteiner, P. P. Pramstaller, K.‐U. Eckardt, A. A. Hicks, F. Kronenberg, the GCKD Investigators

**Affiliations:** ^1^ From the Department of Genetics and Pharmacology Institute of Genetic Epidemiology Medical University of Innsbruck Innsbruck Austria; ^2^ Eurac Research Institute for Biomedicine Affiliated Institute of the University of Lübeck Bolzano Italy; ^3^ Department of Medical Statistics Informatics and Health Economics Medical University of Innsbruck Innsbruck Austria; ^4^ Department of Integrative Physiology University of Colorado Boulder Boulder CO USA; ^5^ Department of Nephrology and Hypertension Friedrich‐Alexander Universität Erlangen‐Nürnberg (FAU) Erlangen Germany; ^6^ Department of Nephrology and Medical Intensive Care Charité – Universitätsmedizin Berlin Berlin Germany

**Keywords:** chronic kidney disease, diabetes, metabolic syndrome, mitochondrial DNA copy number

## Abstract

**Background:**

Mitochondria play an important role in cellular metabolism, and their dysfunction is postulated to be involved in metabolic disturbances. Mitochondrial DNA is present in multiple copies per cell. The quantification of mitochondrial DNA copy number (mtDNA‐CN) might be used to assess mitochondrial dysfunction.

**Objectives:**

We aimed to investigate the cross‐sectional association of mtDNA‐CN with type 2 diabetes and the potential mediating role of metabolic syndrome.

**Methods:**

We examined 4812 patients from the German Chronic Kidney Disease (GCKD) study and 9364 individuals from the Cooperative Health Research in South Tyrol (CHRIS) study. MtDNA‐CN was measured in whole blood using a plasmid‐normalized qPCR‐based assay.

**Results:**

In both studies, mtDNA‐CN showed a significant correlation with most metabolic syndrome parameters: mtDNA‐CN decreased with increasing number of metabolic syndrome components. Furthermore, individuals with low mtDNA‐CN had significantly higher odds of metabolic syndrome (OR = 1.025; 95% CI = 1.011–1.039, *P* = 3.19 × 10^−4^, for each decrease of 10 mtDNA copies) and type 2 diabetes (OR = 1.027; 95% CI = 1.012–1.041; *P* = 2.84 × 10^−4^) in a model adjusted for age, sex, smoking and kidney function in the meta‐analysis of both studies. Mediation analysis revealed that the association of mtDNA‐CN with type 2 diabetes was mainly mediated by waist circumference in the GCKD study (66%) and by several metabolic syndrome parameters, especially body mass index and triglycerides, in the CHRIS study (41%).

**Conclusions:**

Our data show an inverse association of mtDNA‐CN with higher risk of metabolic syndrome and type 2 diabetes. A major part of the total effect of mtDNA‐CN on type 2 diabetes is mediated by obesity parameters.

## Graphical abstract

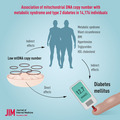

## Introduction

Mitochondria play a pivotal role in the cellular energy metabolism. Their number varies across tissues and cell types, depending on the energy demand of the cell [1]. Mitochondria are dynamic organelles that can alter their morphology in response to changes in the cellular metabolic state. They undergo continuous cycles of fission and fusion that help to maintain a functional balance when cells experience metabolic or environmental stress [2].

The involvement of mitochondrial dysfunction in metabolic syndrome and in type 2 diabetes remains a matter of debate, as reviewed in detail elsewhere [3, 4, 5, 6, 7]. Metabolic syndrome is represented by a variety of metabolic disorders including obesity, hypertension, insulin resistance and dyslipidaemia, and is a precursor of type 2 diabetes, cardiovascular disease and renal disease [8].

Especially, the kidneys have one of the highest densities of mitochondria and a high demand on molecular oxygen, which is mostly metabolized within mitochondria by oxidative phosphorylation for the production of ATP [9]. As thoroughly reviewed recently [10], dysfunctional mitochondria are present in kidney disease and defects include impaired respiratory chain function, structural and networking abnormalities, disrupted cellular signalling and increased reactive oxygen species generation.

Mitochondria have their own genome, the mitochondrial DNA (mtDNA), which is present in multiple copies in each cell and in each mitochondrion. The quantification of mitochondrial DNA copy number (mtDNA‐CN) could be a simple way to assess mitochondrial function and status, and may reflect the level of mtDNA damage [11]. Recent epidemiological studies described an association between mtDNA‐CN and mortality, and cardiovascular disease [12, 13, 14, 15, 16, 17]. Furthermore, several studies have explored the relationship between mtDNA‐CN and type 2 diabetes. However, these investigations are all characterized by small sample sizes and have led to conflicting results [18, 19, 20, 21, 22, 23, 24, 25, 26]. Only few investigations have examined the role of mtDNA‐CN in metabolic syndrome. Some studies observed that reduced mtDNA‐CN correlates with metabolic syndrome components in the general population [27, 28] and that patients with metabolic syndrome have lower mtDNA‐CN than controls [29].

We hypothesized that increased risk of metabolic disorders is associated with decreased mtDNA‐CN. Therefore, we quantified peripheral blood mtDNA‐CN in two large cohorts from Germany and Italy including 4812 patients with moderate chronic kidney disease from the GCKD (German Chronic Kidney Disease) study and 9364 individuals from the population‐based CHRIS (Cooperative Health Research in South Tyrol) study. We assessed the relationship between mtDNA‐CN and both metabolic syndrome and type 2 diabetes in a cross‐sectional analysis. Furthermore, we investigated the potential mediating role of metabolic syndrome components on type 2 diabetes risk.

## Materials and Methods

### GCKD study

The GCKD study is an ongoing prospective multicenter observational cohort study described previously [15]. Briefly, 5217 patients under regular care by nephrologists were enrolled. The inclusion criteria were moderately reduced kidney function defined as estimated glomerular filtration rate (eGFR) 30–60 mL/min/1.73 m^2^ (stage G3, A1–A3) or an eGFR >60 mL/min/1.73 m^2^ in the presence of overt proteinuria (stage G1–G2, A3). The exclusion criteria were non‐Caucasian ethnicity, solid organ or bone marrow transplantation, active malignancy within 24 months prior to screening, heart failure (New York Heart Association Stage IV), legal attendance or inability to provide consent. Information on socio‐demographic factors, medical and family history, medications and health‐related quality of life were obtained by trained personnel through standardized questionnaires. Data are collected and managed using Askimed (https://www.askimed.com) as a cloud‐based web platform for collection and management of Case Report Forms and laboratory data.

### CHRIS study

The CHRIS study is an ongoing longitudinal population‐based study being carried out in South Tyrol (Italy) that aims to investigate the molecular and environmental determinants of common chronic conditions associated with human ageing. A detailed description of the study has been published previously [30]. Blood and urine samples were collected from all participants in the early morning following overnight fasting. Detailed information about medical history and medication was collected by means of interviewer and self‐administered questionnaires. The current project is based on CHRIS data release 3 (16 June 2017) that included 10 518 individuals.

### Informed consent

All participants provided written informed consent, and both studies were carried out in accordance with approved guidelines and in compliance with current national and EU regulations and the Declaration of Helsinki. The GCKD study was approved by the Ethics Committees of all participating institutions and is registered in the National Registry for Clinical Studies (DRKS 00003971). The CHRIS study was approved by the Ethical Committee of the Healthcare System of the Autonomous Province of Bolzano (Protocol No. 21/2011). The project ‘Variazioni del numero di copie del DNA mitocondriale: mutazioni e suscettibilità alle malattie’ (Principal Investigator: Andrew A. Hicks) was approved by the same committee (Protocol No. 10/2016). Analysis of data and samples for this project was authorized by the CHRIS Access Committee (Application No. 69).

### Mitochondrial DNA copy number measurement

Biosamples of both studies were collected in a standardized way and shipped under temperature‐controlled conditions to a central laboratory for routine clinical chemistry and central biobank for future analyses. DNA was extracted from frozen ethylenediaminetetraacetic acid‐treated blood samples using the chemagic Magnetic Separation Module I (PerkinElmer Chemagen Technologie GmbH), an automated magnetic bead‐based method. DNA was available from 4812 and 9364 participants of the GCKD study and CHRIS study, respectively. The quantification of mtDNA‐CN per diploid cell was performed for both studies at the Institute of Genetic Epidemiology of the Medical University of Innsbruck. We applied a duplex quantitative real‐time PCR assay that allows for simultaneous targeting of a single‐copy nuclear gene (*beta‐2 microglobulin*) and the *tRNA‐Leu* gene on the mtDNA as recently described [31]. A plasmid containing both targets was included in all runs in order to correct for interassay variability. This method reduces the interassay variability dramatically, from 21% to 7%. The mtDNA‐CN was calculated using the ∆∆Cq (quantification cycle) method. Two DNA templates included in all the qPCR plates were used to monitor the performance of the assay over the entire study. Interassay mtDNA‐CN coefficients of variation of these two samples analysed in 162 independent experiments were 6.5% and 9.3%. All samples were measured in triplicate. Less than 4% of the samples were excluded from the analysis as they had a coefficient of variation >2% within the triplet reactions. Equal polymerase chain reaction efficiencies for both targets were confirmed in fivefold serial dilutions ranging from 5.0 × 10^6^ to 320 mtDNA copies per reaction. Finally, a total of 4812 samples from the GCKD study and 9364 from the CHRIS study with mtDNA copy number and the other clinical and outcome variables were analysed for this study.

### Outcome definitions

Metabolic syndrome was defined according to the joint statement between several organizations [32]. The metabolic syndrome components are defined as binary variables: (i) waist circumference ≥102 cm in men and ≥88 cm in women in GCKD or body mass index (BMI) above the median of the CHRIS cohort (25.1 kg/m^2^), (ii) fasting triglycerides ≥150 mg/dL (1.69 mmol/L) or ≥175 mg/dL (1.98 mmol/L) for non‐fasting samples and/or drug treatment for elevated triglycerides, (iii) HDL cholesterol <40 mg/dL (1.03 mmol/L) in males and <50 mg/dL (1.29 mmol/L) in females and/or drug treatment for reduced HDL cholesterol, (iv) systolic blood pressure ≥130 mm Hg and/or diastolic blood pressure ≥85 mm Hg and/or antihypertensive drug treatment and (v) fasting glucose ≥100 mg/dL (5.55 mmol/L) or glycosylated haemoglobin HbA_1c_ >42 mmol/mol (6%) and/or drug treatment of elevated glucose. The presence of at least three out of these five components constitutes a diagnosis of metabolic syndrome.

As the GCKD study had not performed measurement of triglycerides in fasting serum samples, we set the threshold for hypertriglyceridaemia at 175 mg/dL [33]. In the CHRIS study, waist circumference data were not available. Therefore, BMI calculated as (weight in kg)/(height in m^2^) was used as a surrogate. Type 2 diabetes was defined as HbA_1c_ ≥ 48 mmol/mol (6.5%) and/or the use of an antidiabetic medication in the GCKD study. In the CHRIS study, type 2 diabetes was defined as self‐reported doctor‐diagnosed diabetes or use of glucose‐lowering drugs or as HbA_1c_ ≥ 48 mmol/mol (6.5%) or fasting glucose ≥126 mg/dL (6.99 mmol/L).

### Statistical analysis

We used pairwise Spearman’s rank‐based coefficient to assess the correlation between mtDNA‐CN and all continuous parameters involved in the metabolic syndrome definition. The effect of mtDNA‐CN on metabolic syndrome was assessed by means of logistic regression models adjusted for age and sex (model 1) and additionally for baseline eGFR, urine albumin‐to‐creatinine ratio (UACR) and smoking status (model 2). We used the same approach to assess the effect of mtDNA‐CN on type 2 diabetes risk by further including all metabolic syndrome components as covariates, except for fasting glucose and HbA_1c_, since they would be part of the outcome definition (model 3). These analyses were conducted for both studies separately and further meta‐analysed using a fixed‐effects model (*I* ^2^ for all models = 0).

To evaluate whether the association between mtDNA‐CN and metabolic syndrome is triggered by specific single components of metabolic syndrome, we also applied the same analysis using each binary metabolic syndrome component as outcome. In addition, we also fitted linear regression models on waist circumference and BMI, as no specific medications are included in the components’ definitions. Since the definition of single components differs slightly between both studies, no meta‐analysis was conducted for the analysis of the single components.

In the CHRIS study, models were further adjusted to account for possible batch and protocol effects [34], as well as for the relatedness structure of the study sample. We fitted a generalized logistic mixed model including the kinship matrix, utilizing the GMMAT R package [35]. The pairwise kinship coefficients were estimated using the ‘kinship2’ R package based on genetic data. All effect estimates refer to a decrease of 10 mtDNA copies.

In both studies separately, we performed a formal mediation analysis for multiple mediators applying the product method approach [36] allowing for categorical mediators [37] to assess the proportion of effect on type 2 diabetes that is mediated by the metabolic syndrome components. Waist circumference and BMI were included as continuous variables. All other components were included as binary variables, since their definitions as metabolic syndrome components also include medications. These models were additionally adjusted for age, sex, smoking, eGFR and log‐transformed UACR. 95% confidence intervals were derived via bootstrap sampling using 1000 iterations.

Since peripheral blood mtDNA‐CN might be influenced by blood cell composition [38], we performed a sensitivity analysis in the CHRIS study where white blood cell and platelet count measurements were available. For this analysis, estimated leucocytes mtDNA‐CN was calculated using a formula that takes into account the contribution of platelets:

$$\text{mtDNA}‐{\text{CN}}_{\text{leucocytes}}\;=\;\;\text{mtDNA}‐{\text{CN}}_{\text{whole blood}}‐K\frac{\text{count of platelets}}{\text{count of leucocytes}}$$

(K is an estimated factor; we used K = 1.1 as described by Hurtado‐Roca and colleagues [38]). All statistical analyses were performed using R 3.3.2 (https://www.r‐project.org), and statistical significance was set at alpha = 0.05.

## Results

Overall, we investigated 14 176 individuals including 4812 from the GCKD study and 9364 from the CHRIS study. The characteristics of the study participants can be found in Table 1. Data for the entire group and stratified for men and women are provided in Table S1 for the GCKD study and in Table S2 for the CHRIS study.

**Table 1:** Characteristics of the participants and distribution of metabolic syndrome components in the GCKD study and the CHRIS study

	GCKD study (*n* = 4812) Mean ± SD or % (*n*) [25th, 50th, 75th percentiles]	CHRIS study (*n* = 9364) Mean ± SD or *n* (%) [25th, 50th, 75th percentiles]
mtDNA‐CN	107.2 ± 36.4	143.8 ± 51.44
Age	60 ± 12 [53, 63, 70]	46 ± 16 [32, 46, 57]
Sex (female)	39.7% (1908)	55.1% (5161)
Waist circumference, cm		
Women	97.4 ± 16.5 [85.0, 96.0, 109.0]	NA
Men	107.8 ± 13.8 [98.0, 107.0, 116.1]	NA
BMI, kg/m^2^		
Women	29.6 ± 6.8 [24.6, 28.6, 33.8]	25.3 ± 4.9 [21.7, 24.2, 27.9]
Men	29.9 ± 5.4 [26.2, 29.0, 32.7]	26.5 ± 4.0 [23.7, 26.0, 28.7]
HDL cholesterol, mg/dL		
Women	59.7 ± 18.7 [46.7, 57.2, 69.8]	67.0 ± 15.5 [56, 66, 76]
Men	46.7 ± 15.4 [36.6, 44.0, 53.4]	54.5 ± 13.5 [45, 53, 62]
Triglycerides, mg/dL	198 ± 125 [117, 168, 240]	104 ± 66 [65, 87, 123]
Total cholesterol, mg/dL	211 ± 53 [176, 207, 239]	211 ± 41 [182, 209, 237]
LDL cholesterol, mg/dL	118 ± 44 [89, 114, 143]	131.45 ± 37.26 [105, 129, 155]
Non‐HLD cholesterol, mg/dL	159 ± 50 [125, 153, 186]	150 ± 41 [120, 146, 176]
Uric acid, mg/dL	7.2 ± 1.9 [5.9, 7.1, 8.3]	5.2 ± 1.4 [4.2, 5.1, 6.1]
Systolic blood pressure, mm Hg	139.5 ± 20.2 [126, 138, 152]	122.2 ± 16.4 [111, 120, 131]
Diastolic blood pressure, mm Hg	79.1 ± 11.7 [71, 79, 87]	78.3 ± 9.4 [72, 77, 84]
Fasting glucose, mg/dL	NA	92.2 ± 13.3 [85, 90, 97]
Glycosylated haemoglobin (HbA1c)	6.3 ± 1.0 [5.7, 6.0, 6.6]	5.56 ± 0.47 [5.3, 5.5, 5.8]
eGFR, mL/min per 1.73 m^2^	49.4 ± 18.2 [37, 46, 58]	91.8 ± 16.3 [81.1, 92.0, 103.1]
UACR, mg/g	432.0 ± 969.6 [9.7, 50.7, 391.0]	15.7 ± 80.0 [4.0, 6.0, 10.6]
Current smokers	15.9% (767)	18% (1687)
Elevated waist circumference or BMI	67.3% (3149)	50.3% (4708)
Elevated blood pressure and/or antihypertensive drug treatment	97.8% (4702)	37.3% (3495)
Reduced HDL cholesterol and/or drug treatment for reduced HDL‐C	36.2% (1730)	11.5% (1077)
Elevated triglycerides and/or drug treatment for elevated triglycerides	49.1% (2352)	14.6% (1367)
Elevated glucose and/or drug treatment of elevated glucose	51% (2437)	17.8% (1666)
Metabolic syndrome	64% (3039)	18.7% (1754)
Type 2 diabetes	35.9% (1726)	4.41% (413)

NA, not available.

### Association of mtDNA‐CN with single metabolic syndrome components

MtDNA‐CN means and standard deviations were 107.2 ± 36.4 in the GCKD study and 143.8 ± 51.4 in the CHRIS study, respectively. A correlation matrix of mtDNA‐CN with each metabolic syndrome parameter on a continuous scale is provided in Figure S1 for each study. Correlations of mtDNA‐CN with metabolic syndrome parameters were direction‐consistent in both studies and were significant with obesity parameters (waist circumference and BMI), diastolic blood pressure, Hb1Ac and HDL cholesterol in the GCKD study and with all parameters in the CHRIS study. In the next step, we applied logistic regression models between mtDNA‐CN and each of the binary metabolic syndrome component definitions, which consider also drug use for blood pressure, HDL cholesterol, triglycerides and glucose. In the age‐ and sex‐adjusted model 1, mtDNA‐CN was significantly associated with the waist circumference component and blood pressure component in the GCKD study and with all components in the CHRIS study (Table 2). In model 2, additionally adjusting for baseline eGFR, UACR and smoking status, a significant association only remained for elevated waist circumference in GCKD, but was observed with all components except glucose in CHRIS. Although not significant, the ORs for the other components were also >1 (except triglycerides in model 2), thus adding to the association with metabolic syndrome. The linear regression model on waist circumference as a continuous variable using the extended adjustment model (model 2) was also significant in the GCKD study (β = 0.3640 for each decrease of 10 mtDNA copies, *P* = 6.86 × 10^−10^).

**Table 2:** Results of logistic regression between mitochondrial DNA copy number and metabolic syndrome components

Metabolic syndrome components	GCKD			CHRIS^a^		
	OR	95% CI	*P*‐value	OR	95% CI	*P*‐value
Model 1: adjusting for age and sex						
Elevated WC (GCKD)/BMI (CHRIS)	1.045	1.027–1.064	**4.81** × **10** ^−^ **^07^**	1.019	1.010–1.028	**3.84** × **10** ^−^ **^05^**
Elevated triglycerides	1.004	0.998–1.020	0.61	1.025	1.012–1.038	**1.29** × **10** ^−^ **^04^**
Reduced HDL cholesterol	1.006	0.990–1.023	0.46	1.028	1.013–1.043	**2.77** × **10** ^−^ **^04^**
Elevated blood pressure	1.054	1.005–1.102	**0.0248**	1.012	1.003–1.022	**0.0139**
Elevated Hb1Ac (GCKD)/fasting glucose (CHRIS)	1.012	0.995–1.029	0.16	1.013	1.001–1.025	**0.0385**
Model 2: adjusting for age, sex, smoking, eGFR and UACR						
Elevated WC (GCKD)/BMI (CHRIS)	1.045	1.027–1.064	**6.69** × **10** ^−^ **^07^**	1.017	1.008–1.026	**1.58** × **10** ^−^ **^04^**
Elevated triglycerides	0.999	0.983–1.015	0.89	1.020	1.007–1.034	**0.0023**
Reduced HDL cholesterol	1.001	0.984–1.018	0.92	1.024	1.009–1.040	**0.0016**
Elevated blood pressure	1.044	0.995–1.093	0.07	1.012	1.002–1.022	**0.0239**
Elevated Hb1Ac (GCKD)/fasting glucose (CHRIS)	1.010	0.992–1.027	0.28	1.010	0.998–1.023	0.09

Odds ratios (OR) are given for decrease of 10 mtDNA copies. The bold values should point out which findings are statistically significant.

eGFR, estimated glomerular filtration rate; Hb1Ac, glycated haemoglobin; UACR, urine albumin‐to‐creatinine ratio; WC, waist circumference.

^a^ CHRIS study additionally adjusted for protocol, batch and kinship matrix (see Supplementary Material).

### Association of mtDNA‐CN with metabolic syndrome

**Fig. 1:**
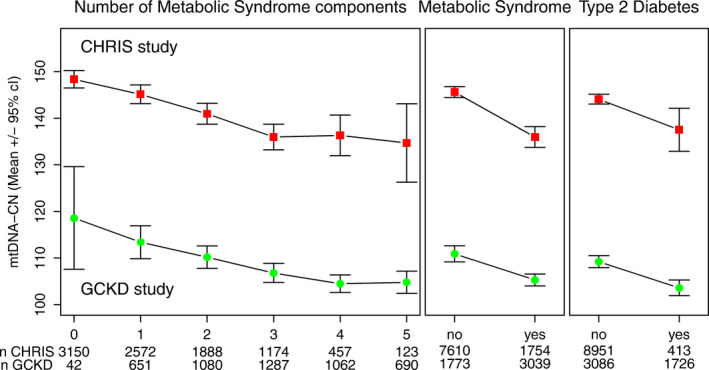
Mean values and corresponding 95% confidence intervals of mtDNA copy numbers for each stratum of numbers of metabolic syndrome components, prevalence of metabolic syndrome and prevalence of type 2 diabetes at baseline in two independent cohorts: GCKD (*n* = 4812) study and CHRIS study (*n* = 9364).

Mean values of mtDNA‐CN are displayed in Fig. 1, revealing that with decreasing mtDNA‐CN the number of metabolic syndrome components increased in both studies (Fig. 1). Logistic regression models adjusting for different sets of variables showed a significant association between a low mtDNA copy number and the odds of metabolic syndrome, with similar results for both studies (Table 3). In the meta‐analysis of both studies, the OR was 1.025 (*P* = 3.19 × 10^−4^) for each 10 mtDNA‐CN decrease in model 2. There was a significant interaction with sex (*P* = 0.0382 in GCKD and *P* < 2 × 10^−16^, in CHRIS), with higher OR in men (OR = 1.046; 95% CI = 1.023–1.070, *P* = 7.67 × 10^−5^, in GCKD and OR = 1.030, 95% CI = 1.013–1.046, *P* = 3.2 × 10^−4^, in CHRIS) than in women (OR = 1.000, 95% CI = 0.976–1.030, *P* = 0.83, in GCKD; and OR = 1.017, 95% CI = 0.998–1.036, *P* = 0.07, in CHRIS). Furthermore, we performed a sensitivity analysis using a formula to calculate mtDNA‐CN taking into account both leucocyte count and platelet count [38]. This additional analysis resulted in only marginal changes in the ORs for metabolic syndrome in CHRIS (model 2, OR = 1.016; 95% CI = 1.003–1.029, *P* = 0.0131).

**Table 3:** Results of logistic regression analysis of mitochondrial DNA copy numbers on metabolic syndrome and type 2 diabetes risk in GCKD and CHRIS study

Adjustment model^b^	GCKD		CHRIS^a^		Meta‐analysis	
	OR [95% CI]	*P*‐value	OR [95% CI]	*P*‐value	OR [95% CI]	*P*‐value
Metabolic syndrome						
Model 1	1.032 [1.014–1.049]	**2.9 × 10^−04^**	1.029 [1.017–1.042]	**0.0015**	1.030 [1.020–1.040]	**1.53** × **10** ^−^ **^09^**
Model 2	1.027 [1.009–1.040]	**0.0025**	1.023 [1.001–1.046]	**0.0070**	1.025 [1.011–1.039]	**3.19** × **10** ^−^ **^04^**
Type 2 diabetes						
Model 1	1.030 [1.012–1.049]	**8.6 × 10^−04^**	1.026 [1.004–1.049]	**0.0206**	1.028 [1.015–1.043]	**4.56** × **10** ^−^ **^05^**
Model 2	1.029 [1.011–1.048]	**0.0016**	1.022 [0.999–1.047]	0.06	1.027 [1.012–1.041]	**2.84** × **10** ^−^ **^04^**
Model 3	1.010 [0.990–1.030]	0.33	1.013 [0.990–1.037]	0.27	1.011 [0.996–1.026]	0.15

Odds ratios (OR) are given for decrease of 10 mtDNA copies. The bold values should point out which findings are statistically significant.

Model 2: as model 1 further adjusted for smoking status, eGFR and UACR

Model 3: as model 2 further adjusted for waist circumference, triglycerides, blood pressure (systolic and diastolic) and HDL cholesterol.

^a^ CHRIS study additionally adjusted for protocol, batch and kinship matrix (see Supplementary Material).

^b^ Model 1: adjusted for age and sex.

### Association of mtDNA‐CN with type 2 diabetes

Low mtDNA‐CN was also significantly associated with higher odds of type 2 diabetes. In model 1, we detected a 2.8% higher odds for diabetes per decrease of 10 mtDNA‐CN (OR = 1.028; *P* = 4.56 × 10^−5^), which remained significant with similar odds in model 2 (OR = 1.027; *P* = 2.84 × 10^−4^). There was no significant interaction with sex (*P* = 0.27 for GCKD and *P* = 0.75 for CHRIS).

### Mediation analysis

**Fig. 2:**
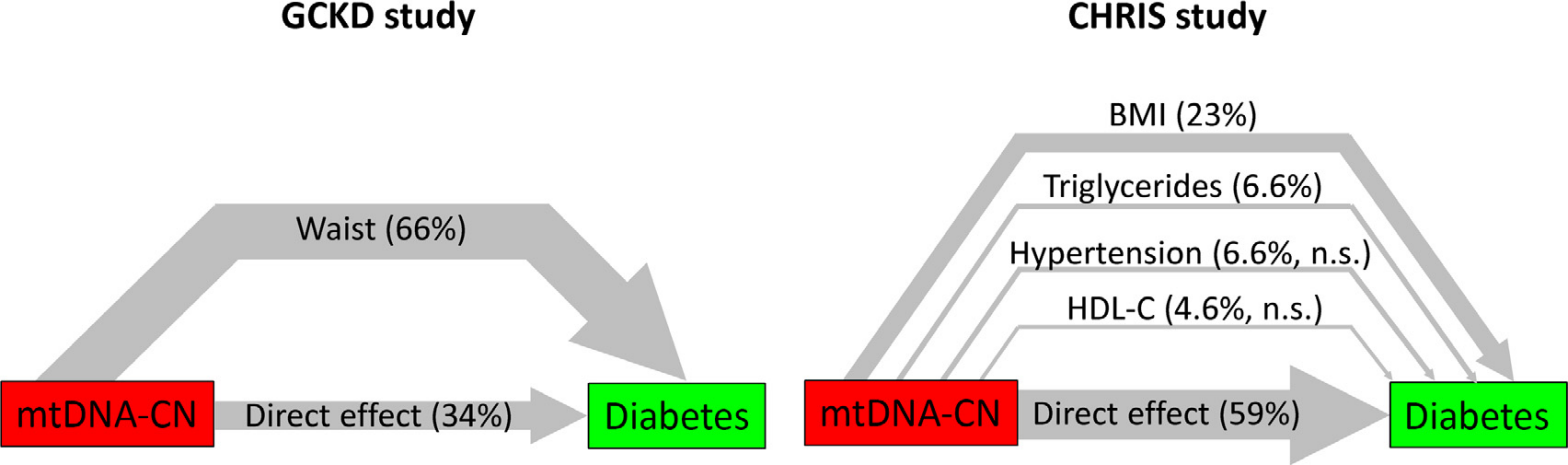
Results of the mediation analysis, depicting the proportion of the total effect of mtDNA copy number on type 2 diabetes mediated by metabolic syndrome components (i.e. indirect effects) and the direct effect in the GCKD study (left panel) and CHRIS study (right panel). The thickness of the lines is proportional to the proportion mediated (n.s.: proportion mediated does not significantly differ from 0).

When we adjusted in a third model for the variables contributing to the metabolic syndrome components, the association between mtDNA‐CN and type 2 diabetes was no longer significant (model 3 in Table 3, *P* = 0.15). This indicates that the effect of mtDNA‐CN on type 2 diabetes risk might be mediated by metabolic syndrome parameters. Providing there is a causal increasing effect of lower mtDNA‐CN on some of the parameters, it might be a reasonable assumption that it is the increase in, for example, obesity or other parameters that increase type 2 diabetes risk, implying that the effect of mtDNA on type 2 diabetes risk is indirect. We therefore performed a formal mediation analysis to evaluate this hypothesis, starting by verifying the requirements for mediation. The first requirement is that mtDNA‐CN is associated with the outcome variable (metabolic syndrome/type 2 diabetes), which was fulfilled (models 1 and 2, Table 3). The second assumption is that mtDNA‐CN is associated with its potential mediators. In model 2, this was only true for waist circumference in GCKD, but was true for all single components in the CHRIS study. Furthermore, mediators have to be associated with type 2 diabetes when adjusted for mtDNA‐CN. This was also fulfilled for waist circumference in GCKD and for four components in CHRIS (Table 2). Therefore, only waist circumference fulfilled the criterion of a potential mediating variable in GCKD, but all four components qualified in the CHRIS study. For comparability, all four components were included in the mediation model also in the GCKD study. 66% of the total effect of mtDNA‐CN on type 2 diabetes was mediated by waist circumference in the GCKD study (Fig. 2 and Table S3). The other metabolic syndrome components did not contribute. In the CHRIS study, 41% of the effect of mtDNA‐CN on type 2 diabetes risk was mediated by the four components in total, with about 23% mediated by BMI, 4.6% by the HDL component and 6.6% by each triglyceride and blood pressure component. The mediating effects of HDL cholesterol and blood pressure did not contribute significantly, though.

## Discussion

The current study including 4812 patients with moderately severe chronic kidney disease and 9364 individuals from a general population sample found mtDNA‐CN to be significantly associated with the prevalence of metabolic syndrome and type 2 diabetes. Furthermore, mediation analysis revealed that a major part of the total effect of mtDNA‐CN on type 2 diabetes was mediated by waist circumference and BMI, respectively.

### Association with metabolic syndrome

To our knowledge, only two small observational studies have previously investigated the association between mtDNA‐CN and metabolic syndrome [28, 29]. The case–control study by Huang et al. including 80 patients with metabolic syndrome and 50 controls revealed that metabolic syndrome patients had lower mtDNA‐CN than control subjects (358 ± 325 vs. 233 ± 112 mtDNA‐CN per cell) [29]. This difference might be a marked overestimation compared with our two cohorts, where we found roughly 5% and 7% difference between subjects with and without metabolic syndrome in GCKD and CHRIS, respectively. Kim et al. in a study with 144 women reported similar findings as in our study [28].

In a longitudinal study with 989 participants, Révész and colleagues showed that mtDNA‐CN at baseline did not predict individual metabolic syndrome component changes during a 10‐year period. Nevertheless, metabolic syndrome diagnosis, waist circumference and fasting glucose levels predicted a 10‐year decrease in mtDNA‐CN [27].

Additional studies explored the association between mtDNA‐CN and various single metabolic parameters, with contradictory results. The majority of these studies [16, 39, 40, 41] with two exceptions [42, 43] reported an inverse association between mtDNA‐CN and metabolic syndrome components. In one of the largest studies performed so far that included only women, the authors demonstrated that mtDNA‐CN had little or no association with 12 cardiometabolic risk factors in 5150 women [42]. This is in line with our sex‐specific analysis, in which we also failed to find an association between mtDNA‐CN and metabolic syndrome in women.

In our study, waist circumference (respectively, BMI) had the strongest association with mtDNA‐CN amongst the metabolic syndrome components. This association is in line with some previous smaller studies [18, 39, 41, 43, 44].

### Association with type 2 diabetes

The relation between mtDNA‐CN with type 2 diabetes and insulin resistance has been explored in several prior studies. These investigations, carried out in a variety of tissues and cell types, are mostly characterized by small sample sizes and inconsistent results: type 2 diabetes was reported to be associated with either an increase [20, 23, 24] or a decrease in mtDNA‐CN [18, 19, 21, 22, 26].

We observed a strong association between mtDNA‐CN and type 2 diabetes in both the GCKD and CHRIS studies, independent of age, sex and kidney function. After adjustment for all components of the metabolic syndrome, the association was no longer significant. We therefore performed a mediation analysis, which revealed that the association of mtDNA‐CN with type 2 diabetes is to a major part mediated by waist circumference in GCKD and by BMI in CHRIS, and to a lesser extent by triglycerides, HDL cholesterol and blood pressure. Despite all similarities in association between mtDNA‐CN and metabolic syndrome and type 2 diabetes between the two studies, results of mediation analysis differed in extent and proportion of mediation. Notably, when taking BMI instead of waist circumference in the GCKD study, percentage mediated was 52%. Therefore, only a small part of the difference can be explained by using different obesity parameters. Since CHRIS is population‐based, but GCKD includes chronic kidney disease patients, prevalences of metabolic syndrome components differ quite substantially, especially for hypertension, HDL cholesterol and triglycerides. It is also a known phenomenon that risk factors differ between chronic kidney disease patients and population‐based cohorts. Therefore, differences have to be expected. However, the overall conclusions of both mediation analyses are quite similar, since in both studies, obesity parameters contribute by far the most. Why waist circumference in the GCKD cohort prominently mediated two third of the association between mtDNA‐CN and diabetes might be explained by the idea that waist circumference is the common pathway of several quite frequent risk factors including other unmeasured metabolic disarrangements in the CKD population, which end finally in diabetes mellitus. Therefore, CKD patients and especially those with a high waist circumference might be closer to a tipping point towards diabetes than the general population sample from the CHRIS study.

It should be noted that it is a prerequisite of a mediation analysis that one assumes a causal relationship. In our study, one would assume that mitochondrial function with mtDNA‐CN as a surrogate marker causally affects both obesity and type 2 diabetes. However, whether the relationship is indeed causal and in which direction cannot be shown in observational epidemiological studies such as ours. Based on the assumption of a causal effect of mtDNA‐CN on metabolic syndrome components, we could then show that mitochondrial dysfunction increases diabetes risk mainly by increasing central obesity.

### Potential mechanism

Although we cannot infer any causal direction of the relationship between mtDNA‐CN and metabolic syndrome from our data, additional evidence supports this assumption. For example, studies in both humans and animals have shown a loss of mtDNA content in metabolic diseases [45]. The pathophysiological mechanism underlying this association has not yet been fully elucidated. However, a ‘metabolic oversupply’ characterized by an excess of energy substrates (e.g. lipids, glucose) not only endorses deterioration of other metabolic syndrome components (e.g. insulin resistance) but also increases cytokine‐mediated inflammation, and induces mitochondrial fragmentation and oxidative stress [27]. This latter is a state that occurs with elevated intracellular levels of reactive oxygen species due to an imbalance between the production of these substances and antioxidant defences. Mitochondrial DNA, owing to its proximity to the electron transport chain, is the prime target of reactive oxygen species [46]. Increased mtDNA damage leads to impaired enzymatic activity and mitochondrial dysfunction, which promotes the development of pathological conditions such as metabolic disorders, ageing and cancer. In contrast, metabolic ‘undersupply’, resulting from caloric restriction and moderate physical activity, increases energy substrate utilization, promotes mitochondrial fusion and limits mtDNA damage [27, 47]. Similarly, it has been shown that hyperglycaemia increases the expression of fission proteins [48] (e.g. dynamin‐related protein 1) and reduces the expression of fusion proteins [47, 49] (e.g. mitofusin 2 and Opa1).

### Strengths and limitations of the study

There are several strengths of our study. We analysed data from 14 176 study participants that include both chronic kidney disease patients and general population individuals. To our knowledge, this is one of the largest investigations of mtDNA‐CN and metabolic disorders. We used the same DNA extraction method for both studies, and the mtDNA‐CN quantification of all samples was performed in a single laboratory using a plasmid normalization approach for correction for interassay variability. This is a significant advantage, since the DNA extraction method can affect tremendously the mtDNA‐CN quantification results [31]. This study has also some limitations: mtDNA‐CN was measured in DNA extracted from total peripheral blood cells. It was not possible to provide data for individual blood cells populations. We acknowledge that other methods could be more appropriate to assess mitochondrial biogenesis and activity, such as the measurement of citrate synthase, oxygen consumption rate or quantification of the mRNA. However, the majority of these methods are performed using tissues, cultured cell or blood that should be drawn and stored following specific conditions. Therefore, all these approaches are not feasible in large epidemiological studies with thousands of participants. Peripheral blood has the advantage that is easily accessible, on the contrary may not reflect mitochondrial function in tissues that contributes to insulin resistance (e.g. skeletal muscle and adipose tissue). However, tissue biopsies are invasive and their use in epidemiological studies is hardly feasible. A further limitation is that waist circumference as a classical component of metabolic syndrome was not measured in the CHRIS study; therefore, BMI was used as a surrogate of waist, which is also used in the American Association of Clinical Endocrinologists (AACE) metabolic syndrome definition [50]. Finally, we consider the use of HbA_1c_ instead of fasting blood glucose as an advantage since HbA1c might be a more stable metabolic index with less preanalytical instability and less biologic variability [51].

The disparity in mtDNA‐CN values between our two studies can be partially explained by the differences in the enrolled populations: chronic kidney disease patients in GCKD and a general population in CHRIS. In the last decade, many studies reported that lower mtDNA‐CN was associated with ageing and various human diseases, including chronic kidney disease [52]. The huge differences in kidney parameters (eGFR and UACR) and age between the CHRIS and GCKD might explain roughly 30% of the difference in mtDNA‐CN values. As discussed previously [31], the measured mtDNA‐CN might be influenced by various factors such as preanalytical conditions including DNA preparation and storage, and standardization of measurement. Therefore, the absolute numbers should not be taken into consideration, and comparisons between studies not measured within the same assay batches should be avoided. We therefore analysed each study separately and then meta‐analysed the results. Despite these differences between the two study populations, it is quite impressive that the main conclusions are valid for both studies.

A further limitation is that chronic kidney disease in the GCKD study could have added complexities that might not simply be reflected or adjusted for by eGFR and albuminuria. We cannot exclude that any potential confounders not reflected in the classical kidney function measures have an impact on bone marrow function and thereby potentially affecting leucocytes and mitochondrial properties.

## Conclusions

In summary, we found lower mtDNA‐CN to be strongly associated with metabolic syndrome and type 2 diabetes both in chronic kidney disease patients and in the general population. The mediation analysis suggested that a major part of the total effect of mtDNA‐CN on type 2 diabetes was mediated by obesity parameters.

## Conflict of interest statement

The authors declare no competing interests.

## Author contribution

**Federica Fazzini:** Data curation (lead); Formal analysis (lead); Methodology (lead); Writing‐original draft (lead); Writing‐review & editing (lead). **Claudia Lamina:** Data curation (supporting); Formal analysis (equal); Supervision (supporting); Visualization (supporting); Writing‐original draft (supporting); Writing‐review & editing (supporting). **Athina Raftopoulou:** Formal analysis (lead); Investigation (lead); Validation (equal); Writing‐original draft (supporting); Writing‐review & editing (supporting). **Adriana Koller:** Data curation (supporting); Investigation (lead); Writing‐review & editing (supporting). **Christian Fuchsberger:** Data curation (supporting); Formal analysis (supporting); Writing‐review & editing (supporting). **Cristian Pattaro:** Data curation (supporting); Funding acquisition (supporting); Resources (lead); Writing‐original draft (supporting); Writing‐review & editing (supporting). **Fabiola M Del Greco:** Data curation (supporting); Formal analysis (supporting); Resources (supporting); Writing‐review & editing (supporting). **Patricia Döttelmayer:** Data curation (supporting); Formal analysis (supporting); Methodology (supporting); Writing‐review & editing (supporting). **Liane Fendt:** Data curation (supporting); Investigation (supporting); Methodology (supporting); Writing‐review & editing (supporting). **Josef Fritz:** Data curation (supporting); Formal analysis (supporting); Methodology (supporting); Software (equal); Writing‐review & editing (supporting). **Heike Meiselbach:** Data curation (lead); Investigation (supporting); Writing‐review & editing (supporting). **Sebastian Schönherr:** Data curation (supporting); Methodology (supporting); Project administration (supporting); Software (lead); Writing‐review & editing (supporting). **Lukas Forer:** Data curation (supporting); Software (lead); Writing‐review & editing (supporting). **Hansi Weissensteiner:** Data curation (supporting); Software (lead); Writing‐review & editing (supporting). **Peter P Pramstaller:** Funding acquisition (supporting); Investigation (supporting); Resources (supporting); Writing‐review & editing (supporting). **Kai‐Uwe Eckardt:** Funding acquisition (supporting); Investigation (supporting); Resources (supporting); Writing‐review & editing (supporting). **Andrew Hicks:** Funding acquisition (supporting); Project administration (supporting); Resources (supporting); Writing‐review & editing (supporting). **Florian Kronenberg:** Conceptualization (lead); Data curation (equal); Formal analysis (supporting); Funding acquisition (lead); Investigation (supporting); Methodology (supporting); Project administration (lead); Resources (lead); Supervision (lead); Validation (supporting); Writing‐original draft (lead); Writing‐review & editing (lead).

## Supporting information

**Figure S1.** Correlation matrix (Spearman correlation coefficient) between mtDNA‐CN and MetS components for GCKD and CHRIS.
**Table S1.** Characteristics of the participants and distribution of metabolic syndrome components in the GCKD study.
**Table S2.** Characteristics of the participants and distribution of metabolic syndrome components in the CHRIS study.
**Table S3.** Mediation analysis results.

## Appendix 1

### Current GCKD Investigators and Collaborators with the GCKD Study are:

**University of Erlangen‐Nürnberg:** Kai‐Uwe Eckardt, Heike Meiselbach, Markus Schneider, Mario Schiffer, Hans‐Ulrich Prokosch, Barbara Bärthlein, Andreas Beck, Detlef Kraska, André Reis, Arif B. Ekici, Susanne Becker, Dinah Becker‐Grosspitsch, Ulrike Alberth‐Schmidt, Birgit Hausknecht, , Anke Weigel;

**University of Freiburg:** Gerd Walz, Anna Köttgen, Ulla T. Schultheiß, Fruzsina Kotsis, Simone Meder, Erna Mitsch, Ursula Reinhard;

**RWTH Aachen University:** Jürgen Floege, Turgay Saritas;

**Charité, University Medicine Berlin:** Elke Schaeffner, Seema Baid‐Agrawal, Kerstin Theisen;

**Hannover Medical School:** Hermann Haller, Jan Menne;

**University of Heidelberg:** Martin Zeier, Claudia Sommerer, Johanna Theilinger;

**University of Jena:** Gunter Wolf, Martin Busch, Rainer Paul;

Ludwig‐Maximilians University of München: Thomas Sitter;

**University of Würzburg:** Christoph Wanner, Vera Krane, Antje Börner‐Klein, Britta Bauer;

**Medical University of Innsbruck,** Institute of Genetic Epidemiology: Florian Kronenberg, Julia Raschenberger, Barbara Kollerits, Lukas Forer, Sebastian Schönherr, Hansi Weissensteiner;

**University of Regensburg,** Institute of Functional Genomics: Peter Oefner, Wolfram Gronwald;

**University of Bonn**, Institute of Medical Biometry, Informatics and Epidemiology, Medical Faculty: Matthias Schmid, Jennifer Nadal.
