# Supplementary material for: Association of mitochondrial DNA copy number with metabolic syndrome and type 2 diabetes in 14 176 individuals
Source: J Intern Med. 2021 Feb 20;290(1):190–202. doi: 10.1111/joim.13242 (PMC8359248; doi:10.1111/joim.13242)
Supplement: Supplementary file 1 — Figure S1. Correlation matrix (Spearman correlation coefficient) between mtDNA‐CN and MetS components for GCKD and CHRIS. Table S1. Characteristics of the participants and distribution of metabolic syndrome components in the GCKD study. Table S2. Characteristics of the participants and distribution of metabolic syndrome components in the CHRIS study. Table S3. Mediation analysis results. [file JOIM-290-190-s001.pdf]

SUPPLEMENTARY MATERIAL

Association of mitochondrial DNA copy number with metabolic syndrome and type 2 diabetes in 14,176 individuals from GCKD and CHRIS studies

Federica Fazzini, PhD [1], Claudia Lamina, PhD [1], Athina Raftopoulou, PhD [2], Adriana Koller, BSc [1], Christian Fuchsberger, PhD [2], Cristian Pattaro, PhD [2], Fabiola M. Del Greco, PhD [2], Patricia Döttelmayer, BSc [1], Liane Fendt, PhD [1], Josef Fritz, PhD [3,4], Heike Meiselbach, PhD [5], Sebastian Schönherr, PhD [1], Lukas Forer, PhD [1], Hansi Weissensteiner, PhD [1], Peter P. Pramstaller, MD [2], Kai-Uwe Eckardt, MD [5,6], Andrew A. Hicks, PhD [2], Florian Kronenberg, MD [1], the GCKD Investigators

[1] Institute of Genetic Epidemiology, Department of Genetics and Pharmacology, Medical University of Innsbruck, Schöpfstrasse 41, Innsbruck, Austria

[2] Institute for Biomedicine, Eurac Research, Affiliated Institute of the University of Lübeck, Galvanistraße 31/Via Galvani 31, Bolzano, Italy

[3] Department of Medical Statistics, Informatics and Health Economics, Medical University of Innsbruck, Innsbruck, Austria

[4] Department of Integrative Physiology, University of Colorado Boulder, 354 UCB, Boulder, CO, USA

[5] Department of Nephrology and Hypertension, Friedrich-Alexander Universität Erlangen-Nürnberg (FAU), Schloßplatz 4, Erlangen, Germany

[6] Department of Nephrology and Medical Intensive Care, Charité – Universitätsmedizin Berlin, Charitépl. 1, Berlin, Germany

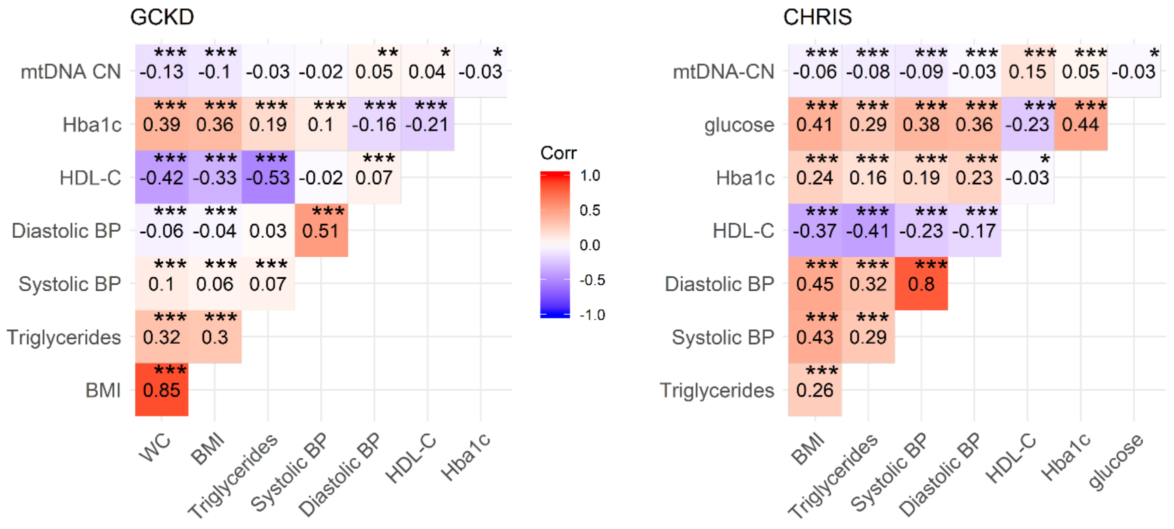

Supplementary Figure S1. Correlation matrix (Spearman correlation coefficient) between mtDNA-CN and MetS components for GCKD (on the left) and CHRIS (on the right). P-values: *<0.05; **<0.01; ***<0.001

Supplementary Table 1. Characteristics of the participants and distribution of metabolic syndrome components in the GCKD study.

	**All participants (n=4,812)**	**Women (n=1,908)**	**Men (n=2,904)**
mtDNA-CN	107.2 ± 36.4 [81.2, 101.9, 126.6]	111.0 ± 37.3 [84.3, 106.2, 130.0]	104.7 ± 35.6 [79.6, 99.4, 124.1]
Age	60 ± 12 [53, 63, 70]	59 ± 13 [51, 63, 69]	61 ± 11 [55, 64, 70]
Sex (female)	39.7% (1,908)		
Waist circumference, cm	103.6 ± 15.8 [93.0, 104, 114.0]	97.4 ± 16.5 [85.0, 96.0, 109.0]	107.8 ± 13.8 [98.0, 107.0, 116.1]
BMI, kg/m^2	29.8 ± 6.0 [25.7, 28.9, 33.1]	29.6 ± 6.8 [24.6, 28.6, 33.8]	29.9 ± 5.4 [26.2, 29.0, 32.7]
HDL cholesterol, mg/dL	51.8 ± 18.0 [39.3, 48.3, 61.2]	59.7 ± 18.7 [46.7, 57.2, 69.8]	46.7 ± 15.4 [36.6, 44.0, 53.4}
Triglycerides, mg/dL	198 ± 125 [117, 168, 240]	178 ± 112 [108, 152, 216]	211 ± 131 [125, 181, 260]
Total cholesterol, mg/dL	211 ± 53 [176, 207, 239]	222 ± 49 [189, 218, 251]	203 ± 53 [169, 199, 230]
LDL cholesterol, mg/dL	118 ± 44 [89, 114, 143]	126 ± 42 [96, 121, 150]	113 ± 44 [84, 108, 137]
Non-HLD cholesterol, mg/dL	159 ± 50 [125, 153, 186]	163 ± 48 [130, 158, 191]	157 ± 52 [122, 151, 183]
Uric acid, mg/dL	7.2 ± 1.9 [5.9, 7.1, 8.3]	6.7 ± 1.9 [5.4, 6.5, 7.7]	7.6 ± 1.9 [6.3, 7.4, 8.6]
Systolic blood pressure,mm Hg	139.5 ± 20.2 [126, 138, 152]	136.3 ± 20.3 [122.0, 134.0, 148.0]	141.6 ± 19.9 [128.0, 140.0, 154.0]
Diastolic blood pressure,mm Hg	79.1 ± 11.7 [71, 79, 87]	79.1 ± 11.4 [71.0, 79.0, 86.0]	79.2 ± 11.7 [71.0, 79.0, 87.0]
Glycosylated haemoglobin (HbA1c)	6.3 ± 1.0 [5.7, 6.0, 6.6]	6.3 ± 1 [5.7, 6.0, 6.4]	6.4 ± 1.0 [5.7, 6.1, 6.7]
eGFR, ml/min per 1.73 m^2	49.4 ± 18.2 [37, 46, 58]	51 ± 19.5 [37, 47, 60]	48.4 ± 17.2 [37.0, 46.0, 56.0]
UACR, mg/g	432.0 ± 969.6 [9.7, 50.7, 391.0]	350.2 ± 875.8 [8.3, 28.9, 229.7]	485.6 ± 1023.0 [11.4, 76.1, 508.1]
Current smokers	15.9% (767)	15.3% (291)	16.4% (476)
Elevated waist circumference	67.3% (3,149)	69.8% (1,298)	65.6% (1,851)
Elevated blood pressure and/or antihypertensive drug treatment	97.8% (4,702)	96.1% (1,830)	98.9% (2,872)
Reduced HDL cholesterol and/or drug treatment for reduced HDL-C	36.2% (1,730)	33.4% (634)	38% (1,096)
Elevated triglycerides and/or drug treatment for elevated triglycerides	49.1% (2,352)	41% (779)	54.6% (1,573)
Elevated glucose and/or drug treatment of elevated glucose	51% (2,437)	47.4% (898)	53.4% (1,539)
**Metabolic syndrome**	**64% (3,039)**	**59.1% (1,117)**	**67.3% (1,922)**
**Type 2 diabetes**	**35.9% (1,726)**	**30.2% (577)**	**39.6% (1,149)**

Data are provided as Mean ± SD or % (n), [25th, 50th, 75th percentile]

Supplementary Table 2. Characteristics of the participants and distribution of metabolic syndrome components in the CHRIS study.

	All participants (n=9,364)	Women (n=5,161)	Men (n=4,203)
mtDNA-CN	143.8 ± 51.4 [110.3, 135.4, 167.4]	146.4 ± 52.5 [112.6, 137.1, 169.9]	140.7 ± 49.9 [107.8, 133.1, 164.5]
Age	46 ± 16 [32, 46, 57]	46 ± 16 [32, 46, 57]	46 ± 16 [32, 46, 58]
Sex (female)	55.1% (5,161)	-	-
BMI, kg/m^2	25.8 ± 4.6 [22.5, 25.1, 28.4]	25.3 ± 4.9 [21.7, 24.2, 27.9]	26.5 ± 4.0 [23.7, 26.0, 28.7]
HDL cholesterol, mg/dL	61.4 ± 15.9 [50, 60, 71]	67.0 ± 15.5 [56, 66, 76]	54.5 ± 13.5 [45, 53, 62]
Triglycerides, mg/dL	104 ± 67 [65, 87, 123]	94± 47 [63, 83, 112]	116 ± 84 [69, 95, 136]
Total cholesterol, mg/dL	211 ± 41 [182, 209, 237]	214 ± 41 [185, 211, 239]	208 ± 42 [182, 209, 237]
LDL cholesterol, mg/dL	131.5 ± 37.6 [105, 129, 155]	129.9 ± 37.0 [104, 127, 154]	133.4 ± 37.4 [107, 132, 158]
Non-HLD cholesterol, mg/dL	150 ± 41 [120, 146, 176]	147 ± 40 [117, 143, 172]	153 ± 42 [120, 146, 176]
Uric acid, unit, mg/dL	5.2 ± 1.4 [4.2, 5.1, 6.1]	4.5 ± 1.1 [3.8, 4.4, 5.1]	6.0 ± 1.2 [5.3, 6.0, 6.8]
Systolic blood pressure, mm Hg	122.2 ± 16.4 [111, 120, 131]	118.5 ± 17.2 [106, 115, 127]	126.7 ± 14.2 [117, 125, 134]
Diastolic blood pressure, mm Hg	78.3 ± 9.4 [72, 77, 84]	76.9 ± 9.4 [70, 76, 82]	79.9 ± 9.1 [73, 79, 86]
Fasting glucose, mg/dL	92.2 ± 13.3 [85, 90, 97]	89.5 ± 12.2 [83, 88, 94]	95.6 ± 13.9 [88, 94, 100]
Glycosylated haemoglobin (HbA1c)	5.6 ± 0.5 [5.3, 5.5, 5.8]	5.6 ± 0.5 [5.3, 5.5, 5.8]	5.6 ± 0.5 [5.3, 5.5, 5.8]
eGFR, ml/min per 1.73 m^2	91.8 ± 16.3 [81.1, 92.0, 103.1]	90.5 ± 16.1 [80.0, 90.5, 101.4]	93.3 ± 16.4 [82.5, 93.9, 104.9]
UACR, mg/g	15.7 ± 80.0 [4.0, 6.0, 10.6]	17.1 ± 74.4 [4.9, 7.6, 12.9]	13.9 ± 87.8 [3.4, 4.6, 7.5]
Current smokers	18.0% (1,687)	17.0% (871)	19.2% (802)
Elevated BMI	50.3% (4,708)	42.5% (2,193)	59.8% (2,515)
Elevated blood pressure and/or antihypertensive drug treatment	37.3% (3,495)	30.6% (1,580)	45.6% (1,915)
Reduced HDL cholesterol and/or drug treatment for reduced HDL-C	11.5% (1,077)	11.2% (579)	11.9% (498)
Elevated triglycerides and/or drug treatment for elevated triglycerides	14.6% (1,367)	10.1% (520)	20.2% (847)
Elevated glucose and/or drug treatment of elevated glucose	17.8% (1,666)	11.0% (568)	26.1% (1,098)
**Metabolic syndrome**	**18.7% (1,754)**	**12.9% (664)**	**25.9% (1,090)**
**Type 2 diabetes**	**4.4% (413)**	**4.3% (224)**	**4.5% (189)**

Data are provided as Mean ± SD or % (n), [25th, 50th, 75th percentile]

Supplementary Table S3. Mediation analysis results

**Proportion mediated in %**	**GCKD**		**CHRIS**	
	Proportion	95% CI	Proportion	95% CI
Total	65.6	[34.1, 227.9]	40.8	[11.5, 293.9]
by waist/BMI	66.0	[35.2, 240.1]	22.9	[5.9, 161.5]
by reduced HDL cholesterol*	-1.1	[-13.02, 6.0]	4.6	[-0.3,41.1]
by elevated triglycerides*	-0.1	[-3.0, 2.7]	6.6	[0.6, 46.2]
by elevated blood pressure *	0.8	[-0.3, 3.5]	6.6	[-4.9, 54.1]

* According to the metabolic syndrome definitions including medications
